# Allograft nephrectomy: a systematic review of immunological consequences and management of immunosuppressants

**DOI:** 10.3389/ti.2026.16661

**Published:** 2026-07-03

**Authors:** Arnaud Del Bello, Anna Goujon, Diana Kassab, Thomas Prudhomme, Alexandre Frontczak, Thibault Culty

**Affiliations:** 1 Nephrology and Organ Transplant Department, CHU de Toulouse, Toulouse, France; 2 Department of Urology, CHU de Rennes, Rennes, France; 3 Department of Methodology, Association Française d’Urologie, Paris, France; 4 Department of Urology, CHU de Toulouse, Toulouse, France; 5 Department of Urology, CHU de Besançon, Besançon, France; 6 Department of Urology, CHU d’Amiens, Amiens, France

**Keywords:** allosensitization, immunosuppression, kidney transplantation, nephrectomy, transplantectomy, kidney embolization, retransplantation

## Abstract

Allograft nephrectomy (AN) is a serious complication of kidney transplantation. Beyond surgical morbidity, AN has important immunological implications. We performed a systematic review to synthesize current evidence on the immunological impact of AN and post-allograft failure immunosuppressive strategies. A systematic review was conducted according to PRISMA guidelines. PubMed/Medline® was searched for studies published from 01/2000 to 03/2026 on transplantectomy indications and surgical techniques. Eligible designs included meta-analyses, randomized trials, and prospective or retrospective studies evaluating the immunological impact of AN and/or immunosuppression management before, during, or after graft removal in adult or pediatric kidney transplant recipients returning to dialysis. Consistent evidence indicates that immunosuppression withdrawal is the principal driver of allosensitization after graft failure. Multiple studies demonstrated marked increases in anti-HLA antibodies following the cessation of immunosuppression, regardless of nephrectomy status. When AN was performed under maintained immunosuppression, its independent effect on sensitization and retransplant outcomes appeared limited. Meta-analyses showed comparable survival after retransplantation, although higher PRA levels, delayed graft function, and acute rejection were more frequent in patients with prior nephrectomy. Allosensitization after graft failure is primarily driven by immunosuppression withdrawal/reduction rather than AN itself. Individualized immunosuppressive management balancing immunological and infectious risks is essential.

## Introduction

Kidney transplantation is the best treatment for patients suffering from end-stage kidney disease, as it provides superior survival, quality of life, and cost-effectiveness while having a lower environmental burden than long-term dialysis [1–3]. Advances in surgical techniques, donor selection, peri-operative management, and immunosuppressive therapies have improved short- and long-term graft outcomes [4, 5]. Nevertheless, graft loss continues to occur in a substantial proportion of transplant recipients, and management of the failed renal allograft represents a major clinical challenge. In this setting, transplant nephrectomy (also referred as “transplantectomy”), defined as the surgical removal of a renal allograft, constitutes a serious complication of kidney transplantation with important clinical, immunological, and prognostic implications.

Transplant nephrectomy may be required at different timepoints after transplantation and for a wide spectrum of indications. In the immediate postoperative period, nephrectomy is most commonly performed due to surgical complications, including arterial or venous thrombosis [6, 7]. At later stages, transplant nephrectomy is frequently indicated after return to dialysis following chronic graft failure, particularly in patients who develop symptomatic rejections (so-called “graft intolerance syndrome,” a condition characterized by graft-related pain, fever, hematuria, anemia, and systemic inflammation reflecting the ongoing immune activation) [8–10], and severe infectious or cancer complications of the graft (such as recurrent pyelonephritis or cancer, depending on the failed graft). More rarely, transplant nephrectomy may be required despite preserved graft function, usually as a consequence of complex vascular, neoplastic, or ureteral complications that are not (or failed to be) amenable to conservative management.

Beyond its indication, transplant nephrectomy is associated with significant morbidity [11]. Infectious complications are among the most frequent and severe adverse events following nephrectomy [12]. Patients undergoing allograft removal often present with multiple predisposing factors, including residual immunosuppression, chronic inflammation related to the failed graft, and malnutrition [13]. Surgical site infections, intra-abdominal abscesses, and sepsis are not uncommon and contribute substantially to early morbidity and mortality [12]. Cardiovascular complications represent another critical concern. Several observational studies have reported increased cardiovascular morbidity and mortality in patients after transplant nephrectomy, although disentangling the independent effects of surgery, graft loss, and dialysis resumption remains challenging [11]. One of the most consequential long-term effects of graft failure and transplant nephrectomy is their role in allosensitization. Removal of the renal allograft has been associated with an increased production of donor specific and non-specific anti-human leukocyte antigen (HLA) antibodies. Several factors, such as the timing of nephrectomy, the duration of graft survival, and the strategy used for immunosuppression withdrawal, have been suspected to influence allosensitization post nephrectomy [6, 14–16]. The immunological consequences of transplant nephrectomy have fueled ongoing debate regarding the optimal management of failed renal allografts. Strategies such as maintaining low-dose immunosuppression have been proposed to mitigate sensitization, but robust evidence remains limited [17, 18]. Furthermore, the balance between reducing immunological risk and minimizing infectious and cardiovascular complications is of paramount importance.

In this systematic review, we aimed to synthesize the evidence regarding the impact of an allograft nephrectomy and the management of immunosuppression.

## Methods

This systematic review was conducted in accordance with the PRISMA (Preferred Reporting Items for Systematic Reviews and Meta-Analyses) guidelines [19]. The protocol for this systematic review was prospectively registered in PROSPERO (International Prospective Register of Systematic Reviews; CRD420251040524).

### Evidence acquisition

A systematic literature search was performed in PubMed/MEDLINE® to identify reports (in French or English) on allograft nephrectomy, published between January 2000 and March 2026, in accordance with PRISMA criteria. The full search strategy is provided in Supplementary Table S1. Study selection was based on the PICOS criteria: Population (P): patients with graft failure after a first kidney transplantation, regardless of severity or age; Intervention (I): allograft transplantectomy (transplant nephrectomy), encompassing different surgical techniques; Comparator (C): other surgical approaches, immunosuppression management strategies, dialysis, or embolization; Outcomes (O): morbidity and mortality (including cardiovascular and immunological impact), adverse events (myocardial infarction, congestive heart failure, sepsis, etc.), time or access to retransplantation, anatomical feasibility for retransplantation, quality of life, and acceptability; and Study design (S): meta-analyses, randomized controlled trials, non-randomized prospective studies, or retrospective studies. Bibliographic data were complemented by literature monitoring (up to June 2024), consultation of international society websites (European Association of Urology, American Urological Association, British Transplantation Society, UK Kidney Association, and American Society of Transplantation), a search for systematic reviews in the Cochrane Library database, and suggestions from the working group. The search strategy was designed to address four predefined clinical questions: (1) the indications for kidney allograft transplantectomy and its optimal timing; (2) the immunological impact of transplantectomy and the management of immunosuppression before, during, and after the procedure; (3) surgical techniques for transplantectomy; and (4) alternatives to transplantectomy, including conservative management strategies. The present article reports the results of the literature selection and evidence synthesis related to question 2, focusing on immunological impact and management of immunosuppressants before, during, and after the allograft nephrectomy.

### Eligibility criteria and study selection

Inclusion and exclusion criteria were predefined prior to the study. Only studies addressing the immunological impact and management of immunosuppressants before, during, and after the allograft nephrectomy were included. Publications deemed ineligible were (i) health economic studies, as they largely depend on country-specific healthcare systems; (ii) case reports, narrative reviews, editorials, letters, or commentaries; (iii) experimental animal or *in vitro* studies; (iv) studies focusing on laboratory technology aspects; (v) studies on clinical practice patterns; and (vi) out-of-scope studies (e.g., not kidney-specific, public health topics not specific to kidney transplantation, immunosuppressive therapy efficacy, transplantation technique, donor nephrectomy techniques in living donors, dialysis modality, retransplantation, or primary non-function related to cancer).

Study selection was initially performed by the methodologist (DK) based on these criteria after abstract screenings. The selection was independently validated by the steering committee (TC, AF, AG, TP, and ADB) and subsequently by the full working group. Full texts of selected publications were then reviewed. Following an initial data extraction by the steering committee, the final set of included studies was confirmed by the steering committee, and any disagreements were resolved through discussion with the working group.

### Quality assessment

The methodological quality of the included studies was evaluated using validated critical appraisal tools appropriate to each study design (e.g., randomized, prospective non-randomized, or retrospective studies), combining criteria derived from the Haute Autorité de Santé (HAS) evidence appraisal framework [20], the STARD (Standards for Reporting Diagnostic Accuracy Studies) checklist [21], and the QUADAS-2 (Quality Assessment of Diagnostic Accuracy Studies-2) tool [22], when applicable. These assessments considered the clinical relevance of the study objectives, representativeness of the selected patients, appropriateness of inclusion and exclusion criteria, relevance of the recruitment period, independence between cohorts, blinding of outcome assessment, identification and adjustment for potential confounding factors, clinical relevance of the compared methods, validity and definition of outcomes, as well as funding sources and conflicts of interest. The level of evidence (LoE) was attributed to each study after taking into account the protocol design and the risk of bias (Supplementary Table S2). The classification of conclusions by LoE (LoE1 is the highest; LoE4 the lowest) was based on the grid proposed by HAS [20]. The risk of bias was assessed independently by two reviewers, and discrepancies were solved by consensus. Randomized and prospective non-randomized studies were prioritized in the analysis, and retrospective studies were included but interpreted with caution. This work was then reviewed by 33 independent experts from all medical and surgical specialties involved in the management of patients with renal graft failure (10 urologists, 4 nephrologists, 4 pediatric nephrologists, 3 immunologists, 3 vascular surgeons, 2 pediatric urologists, 1 pediatric surgeon, 1 infectiologist, and 6 patient representatives). A summary of the methodological analysis of included studies is presented in Supplementary Table S2.

## Results

**FIGURE 1:**
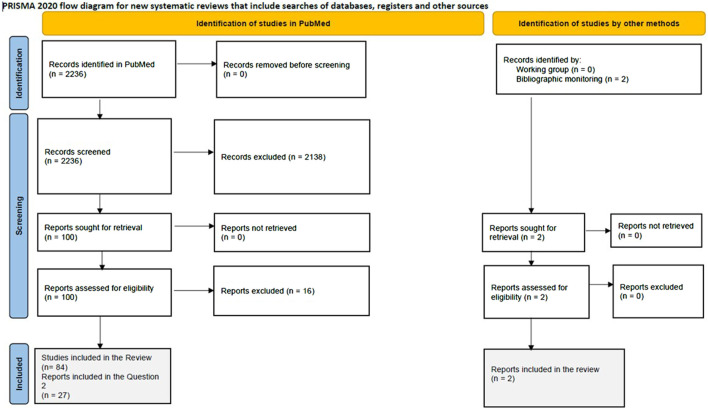
PRISMA flow diagram.

Study selection is detailed in the PRISMA flow diagram (Figure 1). In total, 2,236 records were screened for eligibility and 100 were selected. After full-text review, additional literature checking, and suggestions from the working group, 29 studies were ultimately included for analysis to address the question on “immunological impact and management of immunosuppressants before, during, and after an allograft nephrectomy”: 2 systematic reviews/meta-analyses [23, 24], 9 retrospective comparative studies [15, 25–31], and 18 retrospective observational studies [6, 11, 14, 16, 32–45] summarized in the Table 1.

**TABLE 1:** Brief summary of the included studies.

Study	Design	Included Patients	Objectives	Key results
Naini et al [38]	Retrospective observational	85	To define the prevalence of intolerance syndrome requiring an allograft nephrectomy in patients who stopped immunosuppression after return to dialysis	Intolerance syndrome prevalence: 14% after 47 ± 45 months of follow-up
Knight et al. [16]	Retrospective observational	31	To assess the immunization level before and after an allograft nephrectomy	The mean PRA increased from 33.4 to 75.6 in class I (p < 0.001) and from 38.9 to 60.6 in class II (p = 0.002). This increase was associated with an increase of MESF of DSAs from 33,518 to 121,457 (p < 0.001) in class I and from 45,459 to 126,968 (p < 0.001) in class II. Rejection episodes and a time between dialysis initiation and graft nephrectomy >10 months were associated with a more important increase of PRA (p < 0.001) and MFI DSAs (p = 0.02) after multivariate analysis
Johnston et al [11]	Retrospective observational	19,107 (6,213 with an allograft nephrectomy)	To assess the immunological and non-immunological consequences of an allograft nephrectomy	Analysis of a North American registry (MEDICARE), including 6,213 recipients who required an allograft nephrectomy (32.5%), one-third of which experiencing a return to dialysis during the first year post transplantation. A large majority of patients (89; 3%) required an allograft nephrectomy in the year post return to dialysis. In low sensitized recipients at transplantation (PRA< 30%) allograft nephrectomy was associated with higher PRA at repeat transplantation (p < 0.00001)
Martinez Diaz et al [39]	Retrospective observational	22	To assess immunization following an allograft nephrectomy during the first week post transplantation	Detection of anti-HLA antibodies is observed in 60.8% in class I and 52.2% in class II. DSA were detected in 91.7% of patients with *de novo* anti-class I HLA antibodies and 100% of patients with *de novo* anti-class II HLA.
Lenaers et al [35]	Retrospective observational	32	To assess immunization following an allograft nephrectomy during the first month post transplantation	Sixteen of the thirty-two patients included developed *de novo* DSAs (50%). Anti-class I antibodies were detected in 15 patients, and anti-class II DSA were detected in 10 patients. In multivariate analysis, donor age was an independent predictive factor of *de novo* DSA development (p = 0.05). The median time of *de novo* DSA occurrence was similar regardless the cause of graft failure (immunological vs. non-immunological (67–122 days vs. 9–186 days; p = 0.31)
Sener et al [25]	Retrospective comparative	132 (90 with an allograft nephrectomy)	To compare allosensitization between patients who required an early nephrectomy (<6 months) and those who required a late nephrectomy (>6 months)	A decrease of PRA between dialysis initiation and last follow-up was observed in patients who required an early nephrectomy (n = 39): from 46.2% ± 29.7% to 26.8% ± 28.9%, p = 0.02. Conversely the mean PRA increased in patients who return to dialysis rapidly after transplantation but retained their graft (n = 6) (from 36.2% ± 36.8% (pre-transplant) to 82.8% ± 29.4%, p = 0.02), or those who presented a graft failure after 6 months (n = 51) (from 9.0% ± 11.1% (pre-transplant) to 34.2% ± 30.4% at the time of nephrectomy and to 41.9% ± 30.1% at the time of last follow-up (*p* = 0.002) or without nephrectomy (n = 36): from 4.8% ± 9.0% (pre-transplant) to 17.7% ± 26.2% at time of graft failure and to 27.7% ± 29.4% at the time of last follow-up (*p* = 0.02)
Billen et al. [40]	Retrospective observational	56	To analyze the incidence of *de novo* anti-HLA antibodies after an allograft nephrectomy	During the follow-up post return to dialysis, 81% of previously non-sensitized patients became sensitized to HLA class I, class II, or both (16% before and 84% after graft nephrectomy). HLA class I antibodies were detected in 84% of patients and class II antibodies in 77%. After accounting for the time interval between transplantation and graft nephrectomy (<1 month, 1–6 months, or >6 months), patients who underwent graft nephrectomy between 1 and 6 months had a higher mean number of HLA class II mismatches and more frequently had a history of acute rejection episodes; all patients in this sub-group were DSA-positive. In multivariable analysis, HLA class I DSA positivity was associated with age (OR = 1.06; 95% CI [1.01–1.12]; p = 0.007) and donor type (donation after circulatory death vs. donation after brain death: OR = 32.08; 95% CI [3.4–571.64]; p < 0.0001). In contrast, HLA class II DSA positivity was associated with age (OR = 1.04; 95% CI [1.00–1.08]; p = 0.03) and the number of HLA class II mismatches (OR = 5.31; 95% CI [1.35–20.81]; p = 0.01)
Adeyi et al [41]	Retrospective observational	27	To characterize the profiles of specificities of circulating anti-HLA antibodies detected before and after the nephrectomy	Circulating DSAs were found in 11% before and 97% after graft nephrectomy. In class I, a molecular analysis by HLA matchmaker™ highlight^28^ed a link between anti-HLA non-DSA antibodies and the HLA of the donor
Schrezenmeier et al [42]	Retrospective observational	111	To assess allosensitization after return to dialysis with or without an allograft nephrectomy	38/111 patients (34.2%) already had anti-HLA non-DSA at the time of graft loss 43/111 patients (38.7%) developed *de novo* DSA after graft loss 30/111 patients (27.0%) did not develop dnDSA during the observation period Among relisted patients, the distribution of HLA sensitization between class I and class II antibodies was comparable to that observed in the overall population of patients experiencing graft loss. Notably, patients who underwent graft nephrectomy had rates of *de novo* DSA comparable to those observed in patients without nephrectomy
Schatchner et al [31]	Retrospective comparative	111 (51 with an allograft nephrectomy)	To determine the T cell pre-sensitization after return to dialysis (with or without an allograft nephrectomy)	Kidney transplant recipients with the previous allograft removed showed significantly higher donor-reactive T cells pretransplantation compared with kidney transplant recipients with the previous allograft retained (p = 0.012). Kidney transplant recipients with the previous allograft removed showed significantly higher rates of acute cellular rejection compared with those who retained their allograft [27/51 (53%) vs. 18/60 KTRs (30%); p = 0.019]. Kidney transplant recipients with the previous allograft removed showed significantly inferior death-censored allograft survival (p = 0.022)
Marrari et al [43]	Retrospective observational	65	To analyze allosensitization before and after the nephrectomy	Allo-sensitivity against the HLA A, B loci was present in 64% before and 87% of patients after the nephrectomy (p = 0.0033); for the DRB1 locus: 57% before and 86% after (p = 0.001); for the DRB3/4/5 loci: 65% vs. 78%; p = 0.22; for the DQB locus:76% vs. 87%; p = 0.18)
Lucisiano et al [33]	Retrospective observational	109	To assess the immunological impact of an allograft nephrectomy	An allograft nephrectomy is a predictive and independent factor of anti-HLA DSA development at 24 months post-surgery (HR = 6.12, 95%CI[1.56; 23.93], p = 0.008), decreasing the chances to get a future compatible transplantation. A tacrolimus therapy with trough levels ≥3 ng/mL was a protective factor for allosensitization
Goral et al [6]	Retrospective observational	73	To assess the immunological impact of an allograft nephrectomy	22 patients were tested before and after nephrectomy: 54% of patients presented DSA at both timepoints, and 34% developed *de novo* DSA after the nephrectomy, while the remaining 14% did not develop *de novo* DSA.
Del Bello et al [28]	Retrospective comparative	95	To assess the immunological impact of an allograft nephrectomy	At last follow-up (538 ± 347 days post return to dialysis), the rate of patients with DSA was higher in case of allograft nephrectomy in comparison with patients who retained their transplant (81% vs. 52%, p = 0.02). Similarly, the rate of patients with detectable anti-HLA antibodies (anti-class I 77% vs. 24%, p = 0.001; anti-class II 62% vs. 43%, p = 0.06). An allograft nephrectomy and the A/B mismatches (0 vs. ≥ 1) were independently associated with anti-HLA DSA development
Del Bello et al [14]	Retrospective observational	35	To assess the immunological impact of an allograft nephrectomy after an early graft failure (<3 months)	The rate of patients with at least 1 detectable class I/II DSA was 9.3%, 22.0%, 26.6%, 50%, 56.6%, 56.25%, at transplantation, day 15, and months 1, 3, 6, 9, and 12 post-surgery. The development of *de novo* DSA was not associated with the use of induction therapy
Khakhar et al [36]	Retrospective observational	81	To assess the immunological impact of an allograft nephrectomy	After an allograft nephrectomy the mean PRA increased from 25% to 40% (p < 0.001) at last follow-up, except for the most sensitized patients who presented a stable mean PRA: 89% ± 7% vs. 76% ± 21%; p = 0.05)
Augustine et al [34]	Retrospective observational	119	To assess the immunological impact of an allograft nephrectomy	24 months après return to dialysis, 56% of patients were hypersensitized (class I or II PRA ≥80%). In multivariate analysis, immunosuppression withdrawal was independently associated with the development of hypersensitization (OR = 14,342; 95%IC [2,334–88,144]; p = 0.004). An allograft nephrectomy for intolerance syndrome was required in 41% of patients after immunosuppression withdrawal, vs. 0% in those who maintained their tacrolimus-based immunosuppression (p < 0.001)
Garcia Montemayor [44]	Retrospective observational	146	To assess the immunological impact of an allograft nephrectomy	Among the 72 patients who required an allograft nephrectomy, 41 presented DSA after they returned to dialysis, including 17 for those DSA was detected only after the nephrectomy Fifty-one patients developed DSA during the follow-up (36 [2; 504] weeks: anti-HLA DSA was detected before immunosuppression withdrawal in 21.6%, and after in 78.4% of cases
Kosmoliaptsis et al [45]	Retrospective observational	131	To analyze the relationship between donor mismatches at each HLA locus and the development of HLA Locus-specific antibodies	After return to the waiting-list, the anti-HLA antibody development was independently associated with waitlist duration (>5 vs. < 1year: OR = 8.4; 95%CI [2.58–31.16]; p < 0.001), the number of HLA mismatches (OR = 1.41; 95%IC [1.15–1.74] par mismatch; p < 0.001), and maintenance of a double immunosuppression (OR = 0.2; 95%IC [0.06–0.61]; p = 0.005), but not with allograft nephrectomy All mismatches loci equally contribute to the development of anti-HLA antibodies (HLA-A: OR = 3.2; HLA-B: OR = 3.4; HLA-C: OR = 2.5; HLA-DRB1: OR = 3.5; HLA-DRB3/4/5: OR = 3.9, HLA-DQ/OR = 3.0)
Woodside et al [26]	Retrospective, comparative	186	To analyze the role of immunosuppression withdrawal on incidence of the allograft nephrectomy	An allograft nephrectomy was required in 42% of patients who stopped (n = 60/143) and 23% (n = 11/43) of patients who maintained their immunosuppression therapy (p = 0.026)
Piatosa et al [37]	Retrospective observational	14	To analyze the occurrence of anti-HLA sensitization after graft thrombosis in a pediatric population	Of 14 patients, aged from 4 to 18 years (mean age: 10.7 years), 8 lost their transplant in the first week. The remaining six lost their transplant in the 60 days post transplantation. Anti-HLA antibodies were detected in 12 of the 14 patients (85.7%), including 7 patients with *de novo* DSA (50%), 6 cases with *de novo* anti-class I DSA (42.9%), and one anti-class II (7%)
Nimmo et al [29]	Retrospective comparative	71 (29 with an allograft nephrectomy)	To compare the DSA formation in patients who stopped their immunosuppression, with or with a nephrectomy	All patients who required an allograft nephrectomy stopped their immunosuppression at nephrectomy. An increase of the calculated reaction frequency was observed during the follow-up in both groups, independently of the need to perform a graft nephrectomy. In patients who maintained their transplant, cRF increased from 13% before tapering to 40% at withdrawal and 69% at last follow-up. This was associated with a mean R-CoT (relative chance of transplant) of 54%–46% at 5 years, before and after immunosuppression withdrawal. In patients who required a graft nephrectomy, the mean cRF increased from 31% before immunosuppression, tapering to 69% after withdrawal and 89% at last follow-up. The mean R-CoT decreased from 54% to 42% at 5 years before and after immunosuppression withdrawal
Lachmann [30]	Retrospective comparative	54	To compare allosensitization in patients who returned to dialysis and underwent graft nephrectomy with or without immunosuppression withdrawal, or immunosuppression withdrawal without nephrectomy	Anti-HLA antibodies were similarly detectable in the different settings: 100% of patients after nephrectomy post immunosuppression withdrawal, 100% of patients after nephrectomy under immunosuppression and 92% of patients who underwent immunosuppression only
Freist et al [15]	Retrospective comparative	119	To determine the impact of immunosuppression withdrawal on allosensitization	A lower rate of DSA detection was observed in patients who maintained a calcineurin inhibitor-based immunosuppression (30/52 (57.7%) vs. 52/67 (77.6%), p = 0.02), as well as a lower rate of non-DSA allosensitization (17% vs. 38%, p = 0.03). The rate of graft nephrectomy was similar in both groups (36.5% vs. 49.3%; p = 0.17). In multivariate analysis, the rate of hypersensitized recipients was lower in case of maintenance of immunosuppression
Matignon et al [27]	Retrospective Comparative	63	To compare allosensitization before and after nephrectomy and to assess the role of intravenous immunoglobulins on allosensitization	15/63 patients (24%) required an early graft nephrectomy: All of them received immunosuppression at the surgery, which was stopped at nephrectomy in 8 patients (53%) and tapered during the month for the remaining 7 patients (47%). Among the last 48 late nephrectomies 14 (22%) were asymptomatic, 34 (54%) were due to intolerance syndrome. Patients who required early nephrectomy and asymptomatic recipients presented similar levels of allosensitization, but allosensitization increased during the 3 months post surgery. In patients who received intravenous immunoglobulins after the nephrectomy (n = 10), the rate of anti-HLA non-DSA class I increased (p = 0.03), in contrast with a retrospective control group (n = 13) in which anti-HLA DSA and non-DSA increased in class I, II, and MFI.
Martin et al [32]	Retrospective observational	134	To determine the impact of immunosuppression withdrawal on allosensitization	Allosensitization rate was similar whatever the immunosuppression withdrawal strategy tested (<90, 90–180, >180 days after return to dialysis): cPRA (80.06 vs. 81.21 vs. 85.42; p = 0.66). Similar rates of retransplantation were observed:24/31 [77.4%] vs. 21/35 [60.0%] vs. 22/36 [61.1%]; p = 0.13). A lower rate of nephrectomy was observed in patients who stopped later the immunosuppression (10/42 [23.8%] vs. 7/42 [16.7%] vs. 3/43 [7.0%]; p = 0.01)
Achinger et al	Retrospective observational	18,748	To assess whether allograft nephrectomy is associated with reduced future kidney transplantation	9,374 patients requiring an allograft nephrectomy were matched with 9,374 patients who did not. Patients in the allograft nephrectomy group had a higher chance of undergoing a second transplantation as compared with the comparison group (subdistribution hazard ratio: 1.21; 95%CI 1.15–1.28, p < 0.001), but a higher risk for death (HR:1.09; 95%CI 1.05; 1.13, p < 0.001)
Leal et al	Retrospective observational (data prospectively collected)	90	To assess the impact of prolonging calcineurin inhibitors after graft loss on allosensitization, graft intolerance syndrome, hospitalization rates, mortality	Patients who prolonged calcineurin inhibitors for at least 6 months had significantly lower allograft nephrectomy rates (4.8% vs. 23%, p = 0.015). In multivariate analysis, rapid calcineurin inhibitor withdrawal (OR: 5.9, 95%CI[1.2–32.1), p = 0.04) and acute rejection (OR: 5.6, 95%CI[1.1–27.8), p = 0.04) were independent risk factors for graft intolerance syndrome during the first year Considering HLA sensitization at 1 year post graft failure, allograft nephrectomy (OR 10.9, [1.1; 112.5], p = 0.04), and the number of HLA mismatches (OR: 1.7, 95%CI[1.2–2.5), p = 0.004) were independent predictive factors

Abbreviations: PRA, panel reactive antibody; DSA, Donor-Specific Antibodies; MESF, molecular equivalents of soluble fluorochrome.

### The role of removing the failed graft in the development of allosensitization

The failed graft constitutes a reservoir of donor antigens. In the absence of immunosuppression, ongoing antigen presentation by infiltrating recipient antigen-presenting cells (mainly after late allograft failure) or resident donor-derived cells (in case of early allograft failure) may promote recipient immune activation, leading to antibody formation. The inflammatory milieu surrounding the failing graft may amplify antigen presentation and costimulatory signaling [46–48].

Histopathological data support the role of inflammation. Goral et al [6] reported extensive inflammatory infiltrates in explanted grafts, including plasma cells and tertiary lymphoid structures, which may sustain local antibody production. In such contexts, graft nephrectomy may exacerbate antigen release, immune activation, and *de novo* DSA formation [14, 33].

It was also suggested that the failed graft could adsorb (or regulate) anti-HLA antibody specificities and prevent detection of some anti-HLA antibodies (the so-called “sponge effect”) despite the use of highly sensitive tests [43]. According to this hypothesis, removal of the antigen source could lead to an apparent increase in circulating anti-donor antibodies, as antibodies previously sequestered within the allograft become detectable following graft removal [16, 36]. However, this hypothesis has not been substantiated by direct experimental evidence and is instead supported by indirect clinical observations, including the rapid development of donor-specific antibodies following allograft nephrectomy [16]. However, it should be noted that sera considered as pre-allograft nephrectomy were collected several weeks before the surgery, which could represent a major bias. Recent studies have demonstrated that this effect is probably limited. Indeed, Milongo and colleagues [49] investigated the presence of anti-HLA DSA in both the blood and nephrectomy eluates in 17 patients. They observed that all anti-HLA DSA detected in the nephrectomy eluates were also detected in blood at the time of nephrectomy [49].

### Immunosuppression withdrawal (more than graft nephrectomy in itself) plays a key role in allosensitization after graft failure

While graft nephrectomy has long been considered a major driver of alloimmunization, accumulating evidence suggests that its immunological impact is largely mediated by the management of immunosuppressive therapy rather than by the surgical removal of the graft itself (Table 3). Several studies consistently demonstrated that cessation of immunosuppressive therapy after graft failure is associated with a rapid and marked increase in anti-HLA antibodies, preceding nephrectomy. In a retrospective comparison study involving 69 patients who returned to dialysis and stopped their immunosuppression, Del Bello and colleagues [28] showed that discontinuation of immunosuppression led to a sharp rise in anti-HLA antibodies detection, regardless of whether nephrectomy was subsequently performed (anti-HLA DSA detected in 52% patients without a history of nephrectomy and 81% in those with nephrectomy). Augustine et al [34] further reinforced this concept by demonstrating in a cohort of 119 patients who returned to dialysis that late sensitization after kidney transplant failure was independently associated with immunosuppression weaning, even in the absence of nephrectomy. Patients who discontinued calcineurin inhibitors and antiproliferative agents experienced a significant increase in panel reactive antibodies (PRA) (Median PRA rose from 0% before transplantation to 57% in class I and 63% in class II, 6–24 months post graft failure), indicating broad alloimmune activation. Similar findings were reported by Nimmo et al. [29], who showed that withdrawal of maintenance immunosuppression was the dominant factor influencing the calculated chance of future transplantation, with nephrectomy exerting only a secondary effect. They observed in 41 patients that immunization (expressed by calculated reaction frequency, cRF) rose from 13% pre-immunosuppression weaning to 62% post immunosuppression withdrawal. In the subgroup of 17 patients who underwent graft nephrectomy, the mean cRF rose similarly to others, from 31% pre-immunosuppression wean to 69%. Kosmoliaptsis and colleagues [45] observed that the development of anti-HLA antibodies after return to dialysis in patients listed for another transplantation was independent of graft nephrectomy but associated with waitlist time and maintenance on dual immunosuppression.

The higher allosensitization observed after graft nephrectomy in comparison with immunosuppression weaning only could be related to the surgical intervention in inflammatory conditions and to the high rate of blood transfusion required during or immediately after the surgery [28, 29, 43]. Lucisano et al [33] conducted a retrospective study evaluating the relative contributions of transplantectomy and immunosuppression management to allosensitization in 109 patients. They found that continued immunosuppression (with tacrolimus levels ≥3 ng/mL) was associated with lower rates of *de novo* anti-HLA antibody formation, irrespective of whether transplantectomy was performed. Schachtner et al [31] extended this concept by demonstrating that graft nephrectomy was associated not only with humoral sensitization but also with presensitization at the T-cell level. They investigated the donor-reactive T cell response by using an IFN-γ ELISPOT assay in a cohort of 111 patients who returned to dialysis (51 with a history of graft removal and 60 without). They observed increased frequencies of donor-reactive T cells responses and inferior outcomes after retransplantation in patients with a history of graft removal in comparison with patients without.

### Graft nephrectomy in patients maintained on immunosuppression

Studies focusing on early graft nephrectomy (performed while immunosuppression is ongoing) highlight that sensitization could occur even if the graft nephrectomy is performed only a few minutes after the transplantation. Lenaers et al [35] reported an important (50% of patients) donor-specific alloimmunisation in 32 patients who lost their graft and underwent a nephrectomy during the first 4 months post transplantation. Similarly, Del Bello et al. [14] specifically examined early allograft nephrectomy (<3 months post transplantation) performed under immunosuppression and observed significant anti-HLA immunization (57% of patients after 9 months post nephrectomy). Similarly, when immunosuppression is maintained until nephrectomy in patients with late graft failure, allosensitization is frequent. In a retrospective study of 41 patients (17 with graft nephrectomy, all of whom were maintaining immunosuppression until the surgery), Nimmo and colleagues [29] found high allosensitization (the cRF rose from 31% pre-immunosuppression wean to 69% post-immunosuppression wean and 89% post-immunosuppression cessation). Similarly, Lachmann and colleagues [30] retrospectively compared the allosensitization rate in 28 patients who received a nephrectomy after immunosuppression discontinuation, in 14 patients who received a nephrectomy under immunosuppression (stopped immediately after), and 12 patients who stopped their immunosuppression without nephrectomy. They observed similar allosensitization rates (100% of patients after nephrectomy post immunosuppression withdrawal, 100% of patients after nephrectomy under immunosuppression, and 92% of patients who underwent an immunosuppression only). Matignon and colleagues observed that immunosuppression tapering in the month after the nephrectomy had limited impact on the development of allosensitization, as well as the use of high doses of intravenous immunoglobulins [27]. This could be explained by the persistence of some donor tissue after nephrectomy (in vascular patches). Alloimmunisation could then occur in patients who stop their immunosuppression after the surgery due to these residues [28]. Freist and colleagues [15] observed a lower rate of DSA in patients who maintained a calcineurin inhibitor-based immunosuppression (30/52 (57.7%) vs. 52/67 (77.6%), p = 0.02), as well as a lower rate of non-DSA allo-sensitization (17% vs. 38%, p = 0.03) regardless of the need for an allograft nephrectomy.

As expected, the number of HLA mismatches is an important factor leading to allo-sensitization [28, 40, 45]. All mismatched loci contribute to allosensitization in a similar way [45].

Beyond the formation of anti-HLA DSA, a broad allosensitization is frequently observed after immunosuppression tapering/discontinuation. By using HLA matchmaker™ [28, 41] or recombinant single HLA allele–expressing cell lines [30], several authors demonstrated that a majority of these anti-HLA non-DSA antibodies were directed against donor’s epitopes at the molecular level.

### Graft nephrectomy and impact of retransplantation

The impact of graft nephrectomy on subsequent retransplantation remains a matter of ongoing debate, as its clinical consequences are closely intertwined with alloimmunization and immunosuppression management. In a recent USRDS-based study, Achinger and colleagues observed that patients who underwent an allograft nephrectomy had higher chances of receiving a second transplant (subdistribution HR: 1.21, 95%CI[1.15–1.28, p < 0.001) [50]. When nephrectomy is performed under continued immunosuppression, its independent effect on retransplantation rates appeared limited (Table 2). In an initial meta-analysis of eight studies (1,008 patients), Wang and colleagues [51] did not observe a difference regarding the retransplant outcome (1 year graft survival: OR = 0.74; 95%IC [0.31–1.72]; p = 0.48, 1 year patient survival: OR = 1.60; 95%IC [0.57–4.46]; p = 0.37), acute rejection rate (OR = 1.30; 95%IC [0.89–1.91]; p = 0.17), post-operative complications (OR = 1.51; 95%IC [0.24–9.43]; p = 0.66), or graft function at 1 year (mean ponderate difference: -0.25; 95%IC [-0.52 à 0.03]). Nonetheless, patients with a history of graft nephrectomy present a prolonged time between dialysis and retransplantation (mean ponderate difference in months 11.23; 95%IC [2.47–19.99]; p = 0.01) and increased PRA of more than >10% (OR = 1.62; 95%IC [1.17–2.23]; p = 0.003). Another meta-analysis [13] that included 16 retrospective studies (2,256 patients) reported similar 5-year graft (HR = 1.11, 95%IC [0.89–1.38]; p = 0.37) and patient survival (HR = 0.70; 95%IC [0.45–1.10]; p = 0.12). Nonetheless, patients who underwent graft nephrectomy presented high PRA at retransplantation (%PRA: 39% vs. 35%; OR = 1.83; 95%IC [1.20–2.78]; p = 0.005), delayed graft function (39% vs. 30%; OR = 1.86; 95%IC [1.16–2.98]; p = 0.01), acute rejection (33% vs. 28%; OR = 1.70; 95%IC [1.31–2.22]; p = 0.001), and primary non-function (8% vs. 1.7%; OR = 3.41; 95%IC [1.31–8.89]; p = 0.001).

**TABLE 2:** Summary of meta-analyses regarding retransplantation outcomes.

Study	Included studies/Sample size	Databases searched (n) Reviewers (n)	Intervention	Outcomes	Results
Wang et al. [11]	8 retrospective studies 1,008 patients	5 databases 2 reviewers	Allograft nephrectomy vs. no allograft nephrectomy	After retransplantation 1-year graft survival 1-year patient survival Acute rejection postoperative complications serum creatinine at 1 year	1-year graft survival OR = 0.74; 95% CI [0.31–1.72]; p = 0.48 1-year patient survival OR = 1.60; 95% CI [0.57–4.46]; p = 0.37 Acute rejection OR = 1.30; 95% CI [0.89–1.91]; p = 0.17 Serum creatinine at 1 year WMD: -0.25; 95% CI [-0.52 to 0.03]; p = 0.08
Gavriilidis et al. [9]	16 retrospective studies 2,256 patients	5 databases 2 reviewers	Allograft nephrectomy vs. no allograft nephrectomy	After retransplantation 5-year graft survival 5-year overall survival %PRA Delayed graft function acute rejection Primary non-function graft loss Cold ischemia time	5-year graft survival HR = 1.11, 95% CI [0.89–1.38]; p = 0.37 5-year overall survival HR = 0.70; 95% CI [0.45–1.10]; p = 0.12 %PRA OR = 1.83; 95% CI [1.20–2.78]; p = 0.005 Delayed graft function 39% vs. 30%; OR = 1.86; 95% CI [1.16–2.98]; p = 0.01 Acute rejection OR = 1.70; 95% CI [1.31–2.22]; p = 0.001 Primary non-function OR = 3.41; 95% CI [1.31–8.89]; p = 0.001 Graft loss OR = 1.51; 95% CI [1.09–2.09]; p = 0.01 Cold ischemia time MD = 1.56; 95% CI [0.28–2.85]; p = 0.02
Lin et al. [10]	13 studies 1802 patients	3 databases 2 reviewers	Allograft nephrectomy vs. no allograft nephrectomy	After retransplantation 3–5-year graft survival 5-year patient survival PRA >10% Acute rejection Delayed graft function Cold ischemia time	3-year graft survival OR = 0.48; 95% CI [0.34–0.69]; p < 0.001 5-year graft survival OR = 0.65; 95% CI [0.44–0.97]; p = 0.04 5-year patient survival OR = 1.82; 95% CI [1.14–2.90]; p = 0.01 PRA >10% OR = 3.08; 95% CI [2.08–4.56]; p = 0.000 Acute rejection OR = 1.59; 95% CI [1.21–2.09]; p = 0.0009 Delayed graft function OR = 1.66; 95% CI [1.20–2.03]; p = 0.002 Cold ischemia time MD = 1.84; 95% CI [0.90–2.79]; p = 0.0001

Collectively, these data suggest that graft nephrectomy in itself does not seem to have a detrimental effect on the outcomes of retransplantation.

## Discussion

This systematic review on the immunological impact of AN and post-allograft failure immunosuppressive strategies has been conducted to inform the 2025 French guidelines on Allograft nephrectomy: Indications and surgical techniques [52].

The optimal management of immunosuppressive therapy following graft failure and nephrectomy remains controversial. Limiting the time between graft failure and retransplantation was recently underscored by Kainz and colleagues [53], and allosensitization is a major pitfall for retransplantation. Continuation of low-dose immunosuppression has been proposed to limit alloimmunization, particularly in patients who are candidates for retransplantation. Several studies recently highlighted lowered sensitization rates in [15] patients who maintained immunosuppression after graft failure compared with those who underwent rapid withdrawal [15, 54–56]. García Montemayor et al. [44] similarly observed increased development of donor-specific antibodies after reinitiation of dialysis when immunosuppression was discontinued. Leal and colleagues found that patients who maintained a calcineurin inhibitor after return to dialysis presented a lower rate of graft intolerance syndrome [57]. In a recent meta-analysis of 13 studies comprising 1,531 patients, Fragoso and collegues [54] found that maintaining immunosuppression more than 3 months (with at least two immunosuppressants) was associated with significantly lower odds of graft nephrectomy or embolization (OR:0.41, 95%CI[0.27.0.64]), with no significant impact on hospitalization rates, mortality, or retransplantation. These findings suggest that maintaining some degree of immune modulation may attenuate humoral responses, especially in patients with low baseline immunization. The ideal regimen to propose for patients in dialysis still remains to be identified. The importance of calcineurin inhibitors was highlighted, along with the functioning graft period. Lucisiano and colleagues [33] observed that patients who underwent a nephrectomy under tacrolimus and for whom the treatment was prolonged after the surgery presented a low rate of alloimmunization in comparison with patients who stopped their treatment at nephrectomy. The frequent need for blood transfusions following return to dialysis and in the perioperative period of allograft nephrectomy should also be considered as a potential contributor to HLA sensitization. Given the well-established association between transfusion exposure and alloantibody formation, this factor may partly account for the increase in sensitization observed after graft failure and provides an additional rationale for maintaining immunosuppression in candidates for retransplantation [58, 59]. However, the benefits of prolonged immunosuppression must be weighed against its risks. Woodside et al [26] reported increased rates of infection, fever, and hospitalization in patients who remained on immunosuppressive therapy after graft failure.

This finding supports a tailored approach in which immunosuppression continuation is considered in patients with a realistic prospect of retransplantation and low infectious risk. Dynamic approaches with minimization periods during infection complications and an increase of immunosuppression thereafter need to be assessed. Two studies are now open to assess the effect of controlled immunosuppression to reduce the risk of allosensitization after graft failure: in the PREVSENSI study (NCT06676696), the authors investigate the impact of maintaining a low dose of tacrolimus for 2 years in patients who returned to dialysis for late graft failure (>3 months post transplantation). A nationwide prospective study (TACITE) investigating the impact of maintaining a combination of immunosuppression containing tacrolimus is expected to start soon in France. Finally, a pilot study aiming to reduce allosensitization after an early allograft nephrectomy (RAIPONS study, NCT04779957) suggested that anti-IL6 receptor blockers infusion could limit allosensitization in this setting.

Decision-making regarding immunosuppression after return to dialysis should be individualized, integrating different parameters. The likelihood of retransplantation and baseline sensitization status should be assessed. Highly sensitized patients may derive limited benefit from continued immunosuppression, whereas non- or low sensitized patients may experience substantial harm from immunosuppression withdrawal. This is well illustrated by seminal studies in which PRA before and after transplant nephrectomy remain stable, while an increase was observed in low sensitized patients [36]. Since HLA mismatches burden strongly influences antibody development [45], this parameter should be taken into account. Moreover, the risk of infection should be assessed regularly in immunosuppressed dialysis patients.

Embolization of the failed graft has emerged as a possible less invasive alternative to transplantectomy, particularly in patients with graft intolerance syndrome. Although the immunological impact of embolization in comparison with graft nephrectomy remains to be assessed, we can suppose that embolization could reduce the risk of sensitization by reducing the need for blood transfusion, which is regularly needed after graft nephrectomy.

Finally, our group concluded the following, based on the literature data:

1. After early transplant nephrectomy, the type of induction therapy used does not appear to influence morbidity and mortality [60] or alloimmunization at 1-year post-surgery [14].
2. In patients with late graft failure, systematic transplant nephrectomy before stopping immunosuppression does not seem to prevent anti-HLA immunization after the return to dialysis [29] (LoE4).
3. The rate of anti-HLA immunization (PRA rate >50%) is higher in recipients who underwent transplant nephrectomy than in those who did not [30, 51, 61] (LoE4).
4. After late graft failure, immunosuppressive treatment continuation appears to be well tolerated (at least in the early years after return to dialysis) concerning infection or tumor development [15, 32] (LoE4).

## Conclusion

Our systematic review shows that the immunological impact of graft nephrectomy after kidney allograft failure is largely determined by the timing and manner of immunosuppression withdrawal and by the inflammatory context in which graft removal occurs. Evidence consistently shows that immunosuppression cessation is the principal driver of alloimmunization, often preceding and overshadowing the effects of nephrectomy. When performed while prolonged post-operative immunosuppression is maintained, graft nephrectomy appears to have a limited impact on allosensitization and retransplantation outcomes. Despite the inherent limitations of systematic reviews, we propose that management strategies should therefore prioritize individualized immunosuppression protocols to reduce the need for graft nephrectomy and limit subsequent allosensitization following graft failure.

## Data Availability

The original contributions presented in the study are included in the article/Supplementary Material, further inquiries can be directed to the corresponding author.
