# Supplementary material for: Allograft nephrectomy: a systematic review of immunological consequences and management of immunosuppressants
Source: Transpl Int. 2026 Jul 3;39:16661. doi: 10.3389/ti.2026.16661 (PMC13375601; doi:10.3389/ti.2026.16661)
Supplement: Supplementary file 1 [file Table1.DOCX]

### ***Supplementary Table 1 - Transplantectomy: indications and surgical techniques. Association Française d'Urologie and Société Francophone de Transplantation guidelines – Bibliographic search algorithm - PubMed/Medline® - January 2000 - March 2026, monitoring till April 2026***

| Guidelines |
| --- |
| ("transplantectom*"[Title] OR "graft nephrectomy*"[Title] OR "allograft nephrectomy*"[Title] OR "transplant nephrectomy*"[Title]) OR ("kidney transplantation" [MeSH Terms] OR "kidney transplant*"[Title] OR "renal transplant*"[Title]) AND ("primary graft dysfunction"[Title] OR "primary graft dysfunction"[MeSH Terms] OR "failed transplant*"[Title] OR "failed allograft"[Title] OR "graft failure"[Title] OR "treatment failure"[MeSH Terms] OR "late loss"[Title] OR "graft intolerance"[Title] OR "intolerance syndrome*"[Title] OR "graft rejection/surgery"[MeSH Terms] OR "renal allograft rejection"[Title] OR "reoperation"[MeSH Terms])) OR ("kidney transplantation"[MeSH Terms] OR "kidney transplant*"[Title] OR "renal transplant*"[Title]) AND ("reoperation/methods"[MeSH Terms] OR "retransplantation"[Title] OR "re transplantation"[Title]) AND ("kidney transplantation/adverse effects"[MeSH Terms] OR "kidney failure, chronic/surgery"[MeSH Terms] OR "transplants/surgery"[MeSH Terms] OR "postoperative complications/surgery"[MeSH Terms] OR "donor specific antibodies"[Title] OR "kidney transplantation/methods"[MeSH Terms] OR "surgical technics"[Title] OR "risk factors"[Title] OR "risk factors"[MeSH Terms] OR "time factors"[MeSH Terms] OR "embolization"[Title] OR "embolization, therapeutic/methods"[MeSH Terms] OR "embolotherap*"[Title]) NOT ("liver"[Title] OR "heart"[Title] OR "neoplasms"[MeSH Terms] OR "carcinoma*"[Title] OR "cancer"[Title] OR "malignant epithelial neoplasm*"[Title] OR "tumor*"[Title] OR "animals"[MeSH Major Topic] OR "invertebrate"[Title/Abstract] OR "animal experimentation"[MeSH Major Topic] OR "animal experiment"[Title/Abstract] OR "animal model"[Title/Abstract] OR "animal tissue"[Title/Abstract] OR "animal cell"[Title/Abstract] OR "nonhuman"[Title/Abstract]) AND ("2000/01/01"[Date - Publication] : "2024/03/012"[Date - Publication]) AND (french[Language] OR english[Language]) AND (recommendation*[TI] OR guideline*[TI] OR statement*[TI] OR consensus[TI] OR position paper[TI] OR health planning guidelines[MH] OR practice guideline[PT] OR guideline[PT] OR Consensus Development Conference[PT] OR Consensus Development Conference, NIH[PT]) |
| **Systematic reviews (SR) or meta-analyses (MA), after exclusion of duplicates in the guidelines** |
| ("transplantectom*"[Title] OR "graft nephrectomy*"[Title] OR "allograft nephrectomy*"[Title] OR "transplant nephrectomy*"[Title]) OR ("kidney transplantation" [MeSH Terms] OR "kidney transplant*"[Title] OR "renal transplant*"[Title]) AND ("primary graft dysfunction"[Title] OR "primary graft dysfunction"[MeSH Terms] OR "failed transplant*"[Title] OR "failed allograft"[Title] OR "graft failure"[Title] OR "treatment failure"[MeSH Terms] OR "late loss"[Title] OR "graft intolerance"[Title] OR "intolerance syndrome*"[Title] OR "graft rejection/surgery"[MeSH Terms] OR "renal allograft rejection"[Title] OR "reoperation"[MeSH Terms])) OR ("kidney transplantation"[MeSH Terms] OR "kidney transplant*"[Title] OR "renal transplant*"[Title]) AND ("reoperation/methods"[MeSH Terms] OR "retransplantation"[Title] OR "re transplantation"[Title]) AND ("kidney transplantation/adverse effects"[MeSH Terms] OR "kidney failure, chronic/surgery"[MeSH Terms] OR "transplants/surgery"[MeSH Terms] OR "postoperative complications/surgery"[MeSH Terms] OR "donor specific antibodies"[Title] OR "kidney transplantation/methods"[MeSH Terms] OR "surgical technics"[Title] OR "risk factors"[Title] OR "risk factors"[MeSH Terms] OR "time factors"[MeSH Terms] OR "embolization"[Title] OR "embolization, therapeutic/methods"[MeSH Terms] OR "embolotherap*"[Title]) NOT ("liver"[Title] OR "heart"[Title] OR "neoplasms"[MeSH Terms] OR "carcinoma*"[Title] OR "cancer"[Title] OR "malignant epithelial neoplasm*"[Title] OR "tumor*"[Title] OR "animals"[MeSH Major Topic] OR "invertebrate"[Title/Abstract] OR "animal experimentation"[MeSH Major Topic] OR "animal experiment"[Title/Abstract] OR "animal model"[Title/Abstract] OR "animal tissue"[Title/Abstract] OR "animal cell"[Title/Abstract] OR "nonhuman"[Title/Abstract]) AND ("2000/01/01"[Date - Publication] : "2024/03/012"[Date - Publication]) AND (french[Language] OR english[Language]) AND (metaanalys*[TI] OR meta-analys*[TI] OR meta analysis[TI] OR systematic review*[TI] OR systematic overview*[TI] OR systematic literature review*[TI] OR systematical review*[TI] OR systematical overview*[TI] OR systematical literature review*[TI] OR systematic literature search[TI] OR pooled analysis[TI] OR meta-analysis[PT] OR "Systematic Review" [PT] OR cochrane database syst rev[TA]) NOT (recommendation*[TI] OR guideline*[TI] OR statement*[TI] OR consensus[TI] OR position paper[TI] OR health planning guidelines[MH] OR practice guideline[PT] OR guideline[PT] OR Consensus Development Conference[PT] OR Consensus Development Conference, NIH[PT]) |
| **Prospective randomized studies or not after exclusion of duplicates in the guidelines and SR/MA** |
| ("transplantectom*"[Title] OR "graft nephrectomy*"[Title] OR "allograft nephrectomy*"[Title] OR "transplant nephrectomy*"[Title]) OR ("kidney transplantation" [MeSH Terms] OR "kidney transplant*"[Title] OR "renal transplant*"[Title]) AND ("primary graft dysfunction"[Title] OR "primary graft dysfunction"[MeSH Terms] OR "failed transplant*"[Title] OR "failed allograft"[Title] OR "graft failure"[Title] OR "treatment failure"[MeSH Terms] OR "late loss"[Title] OR "graft intolerance"[Title] OR "intolerance syndrome*"[Title] OR "graft rejection/surgery"[MeSH Terms] OR "renal allograft rejection"[Title] OR "reoperation"[MeSH Terms])) OR ("kidney transplantation"[MeSH Terms] OR "kidney transplant*"[Title] OR "renal transplant*"[Title]) AND ("reoperation/methods"[MeSH Terms] OR "retransplantation"[Title] OR "re transplantation"[Title]) AND ("kidney transplantation/adverse effects"[MeSH Terms] OR "kidney failure, chronic/surgery"[MeSH Terms] OR "transplants/surgery"[MeSH Terms] OR "postoperative complications/surgery"[MeSH Terms] OR "donor specific antibodies"[Title] OR "kidney transplantation/methods"[MeSH Terms] OR "surgical technics"[Title] OR "risk factors"[Title] OR "risk factors"[MeSH Terms] OR "time factors"[MeSH Terms] OR "embolization"[Title] OR "embolization, therapeutic/methods"[MeSH Terms] OR "embolotherap*"[Title]) NOT ("liver"[Title] OR "heart"[Title] OR "neoplasms"[MeSH Terms] OR "carcinoma*"[Title] OR "cancer"[Title] OR "malignant epithelial neoplasm*"[Title] OR "tumor*"[Title] OR "animals"[MeSH Major Topic] OR "invertebrate"[Title/Abstract] OR "animal experimentation"[MeSH Major Topic] OR "animal experiment"[Title/Abstract] OR "animal model"[Title/Abstract] OR "animal tissue"[Title/Abstract] OR "animal cell"[Title/Abstract] OR "nonhuman"[Title/Abstract]) AND ("2000/01/01"[Date - Publication] : "2024/03/012"[Date - Publication]) AND (french[Language] OR english[Language]) AND (random*[TIAB] OR random allocation[MH] OR double-blind method[MH] OR single-blind method[MH] OR cross-over studies[MH] OR randomized controlled trial[PT] OR "Controlled Clinical Trial"[PT] OR multicenter study[PT]) NOT ((metaanalys*[TI] OR meta-analys*[TI] OR meta analysis[TI] OR systematic review*[TI] OR systematic overview*[TI] OR systematic literature review*[TI] OR systematical review*[TI] OR systematical overview*[TI] OR systematical literature review*[TI] OR systematic literature search[TI] OR pooled analysis[TI] OR meta-analysis[PT] OR "Systematic Review" [PT] OR cochrane database syst rev[TA]) OR (recommendation*[TI] OR guideline*[TI] OR statement*[TI] OR consensus[TI] OR position paper[TI] OR health planning guidelines[MH] OR practice guideline[PT] OR guideline[PT] OR Consensus Development Conference[PT] OR Consensus Development Conference, NIH[PT])) |
| **Comparative studies, not controlled clinical trials after exclusion of duplicates in the prospective studies randomized or not, guidelines and SR/MA** |
| ("transplantectom*"[Title] OR "graft nephrectomy*"[Title] OR "allograft nephrectomy*"[Title] OR "transplant nephrectomy*"[Title]) OR ("kidney transplantation" [MeSH Terms] OR "kidney transplant*"[Title] OR "renal transplant*"[Title]) AND ("primary graft dysfunction"[Title] OR "primary graft dysfunction"[MeSH Terms] OR "failed transplant*"[Title] OR "failed allograft"[Title] OR "graft failure"[Title] OR "treatment failure"[MeSH Terms] OR "late loss"[Title] OR "graft intolerance"[Title] OR "intolerance syndrome*"[Title] OR "graft rejection/surgery"[MeSH Terms] OR "renal allograft rejection"[Title] OR "reoperation"[MeSH Terms])) OR ("kidney transplantation"[MeSH Terms] OR "kidney transplant*"[Title] OR "renal transplant*"[Title]) AND ("reoperation/methods"[MeSH Terms] OR "retransplantation"[Title] OR "re transplantation"[Title]) AND ("kidney transplantation/adverse effects"[MeSH Terms] OR "kidney failure, chronic/surgery"[MeSH Terms] OR "transplants/surgery"[MeSH Terms] OR "postoperative complications/surgery"[MeSH Terms] OR "donor specific antibodies"[Title] OR "kidney transplantation/methods"[MeSH Terms] OR "surgical technics"[Title] OR "risk factors"[Title] OR "risk factors"[MeSH Terms] OR "time factors"[MeSH Terms] OR "embolization"[Title] OR "embolization, therapeutic/methods"[MeSH Terms] OR "embolotherap*"[Title]) NOT ("liver"[Title] OR "heart"[Title] OR "neoplasms"[MeSH Terms] OR "carcinoma*"[Title] OR "cancer"[Title] OR "malignant epithelial neoplasm*"[Title] OR "tumor*"[Title] OR "animals"[MeSH Major Topic] OR "invertebrate"[Title/Abstract] OR "animal experimentation"[MeSH Major Topic] OR "animal experiment"[Title/Abstract] OR "animal model"[Title/Abstract] OR "animal tissue"[Title/Abstract] OR "animal cell"[Title/Abstract] OR "nonhuman"[Title/Abstract]) AND ("2000/01/01"[Date - Publication] : "2024/03/012"[Date - Publication]) AND (french[Language] OR english[Language]) AND (clinical trial*[TI] OR comparative stud*[TI] OR versus[TI] OR Clinical Trial[Publication Type:NoExp] OR Comparative Study[PT]) NOT ((random*[TIAB] OR random allocation[MH] OR double-blind method[MH] OR single-blind method[MH] OR cross-over studies[MH] OR randomized controlled trial[PT] OR "Controlled Clinical Trial"[PT] OR multicenter study[PT]) OR (metaanalys*[TI] OR meta-analys*[TI] OR meta analysis[TI] OR systematic review*[TI] OR systematic overview*[TI] OR systematic literature review*[TI] OR systematical review*[TI] OR systematical overview*[TI] OR systematical literature review*[TI] OR systematic literature search[TI] OR pooled analysis[TI] OR meta-analysis[PT] OR "Systematic Review" [PT] OR cochrane database syst rev[TA]) OR (recommendation*[TI] OR guideline*[TI] OR statement*[TI] OR consensus[TI] OR position paper[TI] OR health planning guidelines[MH] OR practice guideline[PT] OR guideline[PT] OR Consensus Development Conference[PT] OR Consensus Development Conference, NIH[PT])) |
| **Observational studies (cohort studies) after exclusion of duplicates in comparative studies, not controlled clinical trials, prospective studies randomized or not, guidelines and SR/MA** |
| ("transplantectom*"[Title] OR "graft nephrectomy*"[Title] OR "allograft nephrectomy*"[Title] OR "transplant nephrectomy*"[Title]) OR ("kidney transplantation" [MeSH Terms] OR "kidney transplant*"[Title] OR "renal transplant*"[Title]) AND ("primary graft dysfunction"[Title] OR "primary graft dysfunction"[MeSH Terms] OR "failed transplant*"[Title] OR "failed allograft"[Title] OR "graft failure"[Title] OR "treatment failure"[MeSH Terms] OR "late loss"[Title] OR "graft intolerance"[Title] OR "intolerance syndrome*"[Title] OR "graft rejection/surgery"[MeSH Terms] OR "renal allograft rejection"[Title] OR "reoperation"[MeSH Terms])) OR ("kidney transplantation"[MeSH Terms] OR "kidney transplant*"[Title] OR "renal transplant*"[Title]) AND ("reoperation/methods"[MeSH Terms] OR "retransplantation"[Title] OR "re transplantation"[Title]) AND ("kidney transplantation/adverse effects"[MeSH Terms] OR "kidney failure, chronic/surgery"[MeSH Terms] OR "transplants/surgery"[MeSH Terms] OR "postoperative complications/surgery"[MeSH Terms] OR "donor specific antibodies"[Title] OR "kidney transplantation/methods"[MeSH Terms] OR "surgical technics"[Title] OR "risk factors"[Title] OR "risk factors"[MeSH Terms] OR "time factors"[MeSH Terms] OR "embolization"[Title] OR "embolization, therapeutic/methods"[MeSH Terms] OR "embolotherap*"[Title]) NOT ("liver"[Title] OR "heart"[Title] OR "neoplasms"[MeSH Terms] OR "carcinoma*"[Title] OR "cancer"[Title] OR "malignant epithelial neoplasm*"[Title] OR "tumor*"[Title] OR "animals"[MeSH Major Topic] OR "invertebrate"[Title/Abstract] OR "animal experimentation"[MeSH Major Topic] OR "animal experiment"[Title/Abstract] OR "animal model"[Title/Abstract] OR "animal tissue"[Title/Abstract] OR "animal cell"[Title/Abstract] OR "nonhuman"[Title/Abstract]) AND ("2000/01/01"[Date - Publication] : "2024/03/012"[Date - Publication]) AND (french[Language] OR english[Language]) AND (cohort*[TI] OR longitudinal stud*[TI] OR follow-up stud*[TI] OR prospective stud*[TI] OR retrospective stud*[TI] OR cohort studies[MH] OR longitudinal studies[MH] OR follow-up studies[MH] OR prospective studies[MH] OR Retrospective Studies[MH] OR "Observational Study" [Publication Type]) NOT ((clinical trial*[TI] OR comparative stud*[TI] OR versus[TI] OR Clinical Trial[Publication Type:NoExp] OR Comparative Study[PT]) OR (random*[TIAB] OR random allocation[MH] OR double-blind method[MH] OR single-blind method[MH] OR cross-over studies[MH] OR randomized controlled trial[PT] OR "Controlled Clinical Trial"[PT] OR multicenter study[PT]) OR (metaanalys*[TI] OR meta-analys*[TI] OR meta analysis[TI] OR systematic review*[TI] OR systematic overview*[TI] OR systematic literature review*[TI] OR systematical review*[TI] OR systematical overview*[TI] OR systematical literature review*[TI] OR systematic literature search[TI] OR pooled analysis[TI] OR meta-analysis[PT] OR "Systematic Review" [PT] OR cochrane database syst rev[TA]) OR (recommendation*[TI] OR guideline*[TI] OR statement*[TI] OR consensus[TI] OR position paper[TI] OR health planning guidelines[MH] OR practice guideline[PT] OR guideline[PT] OR Consensus Development Conference[PT] OR Consensus Development Conference, NIH[PT])) |
| **Other studies after exclusion of duplicates in observational studies (cohort studies), comparative studies, not controlled clinical trials, prospective studies randomized or not, guidelines and SR/MA** |
| ("transplantectom*"[Title] OR "graft nephrectomy*"[Title] OR "allograft nephrectomy*"[Title] OR "transplant nephrectomy*"[Title]) OR ("kidney transplantation" [MeSH Terms] OR "kidney transplant*"[Title] OR "renal transplant*"[Title]) AND ("primary graft dysfunction"[Title] OR "primary graft dysfunction"[MeSH Terms] OR "failed transplant*"[Title] OR "failed allograft"[Title] OR "graft failure"[Title] OR "treatment failure"[MeSH Terms] OR "late loss"[Title] OR "graft intolerance"[Title] OR "intolerance syndrome*"[Title] OR "graft rejection/surgery"[MeSH Terms] OR "renal allograft rejection"[Title] OR "reoperation"[MeSH Terms])) OR ("kidney transplantation"[MeSH Terms] OR "kidney transplant*"[Title] OR "renal transplant*"[Title]) AND ("reoperation/methods"[MeSH Terms] OR "retransplantation"[Title] OR "re transplantation"[Title]) AND ("kidney transplantation/adverse effects"[MeSH Terms] OR "kidney failure, chronic/surgery"[MeSH Terms] OR "transplants/surgery"[MeSH Terms] OR "postoperative complications/surgery"[MeSH Terms] OR "donor specific antibodies"[Title] OR "kidney transplantation/methods"[MeSH Terms] OR "surgical technics"[Title] OR "risk factors"[Title] OR "risk factors"[MeSH Terms] OR "time factors"[MeSH Terms] OR "embolization"[Title] OR "embolization, therapeutic/methods"[MeSH Terms] OR "embolotherap*"[Title]) NOT ("liver"[Title] OR "heart"[Title] OR "neoplasms"[MeSH Terms] OR "carcinoma*"[Title] OR "cancer"[Title] OR "malignant epithelial neoplasm*"[Title] OR "tumor*"[Title] OR "animals"[MeSH Major Topic] OR "invertebrate"[Title/Abstract] OR "animal experimentation"[MeSH Major Topic] OR "animal experiment"[Title/Abstract] OR "animal model"[Title/Abstract] OR "animal tissue"[Title/Abstract] OR "animal cell"[Title/Abstract] OR "nonhuman"[Title/Abstract]) AND ("2000/01/01"[Date - Publication] : "2024/03/012"[Date - Publication]) AND (french[Language] OR english[Language]) NOT (letter[PT] OR editorial[PT] OR news[PT] OR comment[PT]) NOT ((cohort*[TI] OR longitudinal stud*[TI] OR follow-up stud*[TI] OR prospective stud*[TI] OR retrospective stud*[TI] OR cohort studies[MH] OR longitudinal studies[MH] OR follow-up studies[MH] OR prospective studies[MH] OR Retrospective Studies[MH] OR "Observational Study" [Publication Type]) OR (clinical trial*[TI] OR comparative stud*[TI] OR versus[TI] OR Clinical Trial[Publication Type:NoExp] OR Comparative Study[PT]) OR (random*[TIAB] OR random allocation[MH] OR double-blind method[MH] OR single-blind method[MH] OR cross-over studies[MH] OR randomized controlled trial[PT] OR "Controlled Clinical Trial"[PT] OR multicenter study[PT]) OR (metaanalys*[TI] OR meta-analys*[TI] OR meta analysis[TI] OR systematic review*[TI] OR systematic overview*[TI] OR systematic literature review*[TI] OR systematical review*[TI] OR systematical overview*[TI] OR systematical literature review*[TI] OR systematic literature search[TI] OR pooled analysis[TI] OR meta-analysis[PT] OR "Systematic Review" [PT] OR cochrane database syst rev[TA]) OR (recommendation*[TI] OR guideline*[TI] OR statement*[TI] OR consensus[TI] OR position paper[TI] OR health planning guidelines[MH] OR practice guideline[PT] OR guideline[PT] OR Consensus Development Conference[PT] OR Consensus Development Conference, NIH[PT])) |
