# Supplementary material for: Allograft nephrectomy: a systematic review of immunological consequences and management of immunosuppressants
Source: Transpl Int. 2026 Jul 3;39:16661. doi: 10.3389/ti.2026.16661 (PMC13375601; doi:10.3389/ti.2026.16661)
Supplement: Supplementary file 2 [file Table2.DOCX]

**Supplemental 2: Methodological analysis of the included studies**

| Reference | Level of evidence (LoE) | Study design | Critical analysis of original studies^[[1]](#footnote-1)^ |
| --- | --- | --- | --- |
| Del Bello et al. 2012^1^ | 4 | Retrospective comparative | **Retrospective comparative study investigating a relatively large cohort. Exclusion criteria are presented and relevant. Objectives are clear.**  **Anti-HLA antibodies were detected by the luminex single antigen assay (but without pro-zone effect determination) in both groups.** |
| Marrari et al. 2010^2^ | 4 | Retrospective comparative | **Retrospective study from a small cohort of patients. Immunosuppression received (before or after the nephrectomy) by patients is not mentionned. Pre and post nephrectomy sera were assessed with very very large intervals regarding the timepoint of interest.** |
| Del Bello et al. 2012^3^ | 4 | Retrospective observational | Retrospective study from a small single center study. Inclusion, exclusion criteria and objectives are clearly mentioned. |
| Lucisano et al. 2019^4^ | 4 | Retrospective observational | **Retrospective study including patients with different regimen of immunosuppression before the nephrectomy, and the management of tacrolimus tapering changed with time, without adjustment. Data regarding the relisting and retransplantation are not mentioned.** |
| Goral et al. 2014^5^ | 4 | Retrospective observational | **Small cohort. Histological analysis refers to Banff Classification. Few details regarding the immunosuppression in the late nephrectomy group.** |
| Khalhar et al. 2003^6^ | 4 | Retrospective observational | Relatively large study (n=89). Included patients are in line with objectives of the study. Inclusion criteria are clearly mentioned. The criteria assessed are relevant, but the measuring methods are not appropriate given the absence of a maximum time limit between nephrectomy and pre- or post-immunological analysis, which leads to considerable discrepancies. These results need to be confirmed in a multicenter study. |
| Augustine et al. 2012^7^ | 4 | Retrospective observational | In this retrospective, non-comparative study, the patient population recruited corresponds to the study objective: patients followed up at the center who required graft removal between 1998 and 2010 after a transplant that had functioned for at least one month (7 years on average), and for whom cPRA follow-up was available at 6 and 24 months post-return to dialysis. The exclusion criteria, inclusion period, and follow-up duration are clinically relevant. All relevant variables were considered in the multivariate analysis. However, the study is single-center; the results need to be confirmed in a larger independent cohort. |
| Garcia Montemayor et al. 2015^8^ | 4 | Retrospective observational | In this small, descriptive, non-comparative retrospective study, the inclusion criteria are unclear and it is unclear whether patients without antibodies prior to returning to dialysis were excluded. We note major variations in the management of immunosuppressants. The endpoint was poorly defined. The objective of the study is not explicit. The method used to measure DSA is not described. Other immunization factors were not taken into account in the multivariate analysis. |
| Kosmoliaptsis et al. 2014^9^ | 4 | Retrospective observational | In this retrospective comparative study, the patient population recruited corresponds to the study objective. The exclusion criteria are relevant. |
| Woodside et al. 2014^10^ | 4 | Retrospective comparative | The exclusion criteria, inclusion period, and variables included in the multivariate analysis are clinically relevant. The limitations are mainly related to the small number of patients maintaining immunosuppression and the different reasons for maintaining it (many combined pancreas and kidney transplants, with functional pancreas in the group maintaining immunosuppression). The study is single-center; the results need to be confirmed in a larger independent cohort. |
| Piatosa et al. 2006^11^ | 4 | Retrospective observational | This is a small, specific, retrospective, descriptive, non-comparative study of the pediatric population. The objective of the study is unclear, but it appears to describe the role of rejection-related DSA in the occurrence of early graft loss. However, as there is no comparator group, it is not possible to answer the question. Furthermore, the rationale for focusing on the pediatric population is not explained. |
| Nimmo et al. 2018^12^ | 4 | Retrospective comparative | the patient population recruited corresponds to the objective. The two groups compared are homogeneous, but the sample size is small and the comparator group is biased in terms of the indication for nephrectomy and the time between nephrectomy and last follow-up, compared to the last follow-up only in the group without nephrectomy. We note the absence of statistical comparison between the “nephrectomy” group and the “no nephrectomy” group. |
| Freist et al. 2021^13^ | 4 | Retrospective comparative | This is a retrospective study with a fairly large patient population. Objectives, Inclusion and exclusion criteria are clearly defined. |
| Matignon et al. 2016^14^ | 4 (for the retrospective part)  2 (for the prospective part) | Retrospective and prospective  Comparative | **Two studies in a single article with a very small sample size, the results of which are compared with a historical cohort. The first part of the study is retrospective (n=63). It involves patients who underwent early transplantectomy (within 6 months of transplantation) or late transplantectomy (more than 6 months). Late transplantectomies were then divided into two groups: asymptomatic vs. graft intolerance. For the second part of the study, all patients who were scheduled to undergo transplantectomy without preoperative DSA were prospectively included (n=10) and compared with a historical cohort of patients without pre-transplantectomy DSA.** |
| Martin et al . 2021^15^ | 4 | Retrospective comparative | **Study based on a large cohort. The inclusion and exclusion criteria are clear. Follow-up is important. The objectives are well defined.** |
| Schatner et al 2018^16^ | 4 | Retrospective comparative | **small sample size. There was a difference in the management of immunosuppressive treatments between anuric patients and patients with preserved diuresis, but no subgroups analysis. The average time between transplantectomy and re-transplantation was 5 years, but the study did not specify the time between return to dialysis and re-transplantation*.*** |
| Lachmann et al 2016^17^ | 4 | Retrospective comparative | **Small sample size study (with 3 different groups of 28, 14, and 12 patients). Inclusion and exclusion criteria, as well as objectives are clear. Time between sera screened for analysis, management of immunosuppression and the time point of interest are heterogeneous according to the group (**nephrectomy with concomitant withdrawal of IS, nephrectomy in patients off immunosuppression, and withdrawal IS without nephrectomy). |
| Knight et al. 2011^18^ | 4 | Retrospective observational | In this single-center, retrospective, non-comparative study, the patient population recruited corresponds to the study objective. The exclusion criteria, inclusion period, and follow-up duration are clinically relevant, but the criteria measured are outdated and inconsistent (several methods of DSA detection, PRA, etc.). The measurement methods are not appropriate because the maximum time between nephrectomy and pre- or post-immunological analysis is not specified, which leads to considerable discrepancies. Not all relevant variables were considered in the multivariate analysis, in particular other immunizing events such as transfusion. The results are difficult to interpret given the small sample size and the variability in the time between the pre- and post-nephrectomy immunological analysis and the nephrectomy. |
| Naini et al. 2008^19^ | 4 | Retrospective observational | **Small sample size study. Multiples biases regarding exclusion criteria linked to retransplantation and death.** |
| Lenaers et al^20^ | 4 | Retrospective observational | In this retrospective, non-comparative study, the patient population recruited corresponds to the study objective. The exclusion criteria, inclusion period, and criteria measured are clinically relevant. However, the study is single-center, the sample size is small, and the measurement methods are not appropriate, as there is no maximum time limit between nephrectomy and pre- or post-immunological analysis, which leads to considerable discrepancies. Not all relevant variables were considered in the multivariate analysis, in particular other immunizing events such as transfusion. |
| Billen et al.^21^ | 4 | Retrospectiveobservational | In this small, descriptive, non-comparative retrospective study, the patient population recruited corresponds to the study objective. The two groups are comparable. The exclusion criteria and follow-up period are clinically relevant. All relevant variables were considered in the multivariate analysis. |
| Sener et al^22^ | 4 | Retrospective comparative | In this retrospective comparative study, the patient population recruited corresponds to the study objective and the exclusion criteria are relevant. All relevant variables were considered in the multivariate analysis. However, the two groups compared are not homogeneous since the indications for nephrectomy differed between the groups. The separation between early transplantectomy at 6 months vs. later and maintenance of immunosuppression is not justified. |
| Adeyi et al^23^ | 4 | Retrospective observational | In this small, descriptive, retrospective, non-comparative study, no inclusion or exclusion criteria are specified. The median time since graft loss was 1401 [12-4448] days, a wide range that highlights heterogeneity. The total number of graft losses at the center during the study period is not mentioned. |
| Martinez Diaz^24^ | 4 | Retrospective observational | In this small, descriptive, non-comparative retrospective study, the patient population recruited does not correspond to the objective given the absence of a comparator arm of patients who did not lose their graft in the first week. The exclusion criteria are not relevant since exclusion beyond day 8 was not justified, and not clear (notion of hypersensitization not defined). The measurement methods are not appropriate given the absence of a positivity threshold definition. The objective of the study is unclear but appears to focus on the detection of DSAs after nephrectomy and their persistence over time, but no follow-up is planned in the study. |
| Schrezenmeier^25^ | 4 | Retrospective observational | **In this relatively large descriptive study, inclusion and exclusion criteria are clearly mentioned, and objectives are well defined. The measurement method is not appropriate since detection of DSA was performed with different methods according to the period of graft failure.** |
| Achinger^26^ | 4 | Retrospective observational | **Retrospective study from the USRDS. The variables included in the algorithm used for the propensity score matching were not selected with a rigorous literature based selection (For instance, the PSM omitted immunological and surgical parameters).**  **Cause and effect relationship between allograft nephrectomy and retransplantation cannot be assessed.** |
| Leal^27^ | 4 | Retrospective study of prospectively acquired data observational | In this relatively large descriptive study involving 90 patients, the inclusion and exclusion criteria are clearly stated, and the study objectives are well defined.  However, the rationale for group allocation (rapid versus prolonged immunosuppression withdrawal) remains unclear, as treatment decisions were left to the discretion of the treating clinician. In addition, graft intolerance syndrome, which was included as part of the primary outcome, was not clearly defined. |
| *Patient population recruited in line with the objective? Homogeneous in the two groups compared? Relevant exclusion criteria? Patient sample size? Calculation of the number of subjects required based on the primary endpoint? Power not adapted to sample size (small) not suitable, for example, for an equivalence study or multiple secondary endpoints? Lost to follow-up not specified or too high in number? Is the inclusion period clinically relevant? Primary endpoint defined? Relevant criteria measured? Validated? Calculation of the number of subjects required based on the primary endpoint? Multiple secondary endpoints? Appropriate measurement methods? Adequacy of the study protocol for the question addressed? Is the reference test validated and appropriate (gold standard)? Is the test performed as part of the usual diagnostic strategy (sequence)? Is the threshold used clinically relevant? Definition of a priori/a posteriori analyses (subgroup analysis or post-hoc analysis)? Have all relevant variables been considered in the multivariate analysis? Intention-to-treat analysis? Number of patients lost to follow-up? Clinically relevant follow-up period? Results: statistical significance/clinical relevance/relevant measurement time/applicability (population, age, etc.)/multiple secondary criteria?* | | | |

Abbreviation: ART, assisted reproduction technologies; NA, not applicable; WHO, World Health Organization, DSA Donor Specific Antibodies, IS, Immunosuppression.

**References**

1. Del Bello A, Congy-Jolivet N, Sallusto F, et al. Donor-specific antibodies after ceasing immunosuppressive therapy, with or without an allograft nephrectomy. *Clinical Journal of the American Society of Nephrology*. 2012;7(8). doi:10.2215/CJN.00260112

2. Marrari M, Duquesnoy RJ. Detection of donor-specific HLA antibodies before and after removal of a rejected kidney transplant. *Transpl Immunol*. 2010;22(3-4):105-109. doi:10.1016/J.TRIM.2009.12.005

3. Del Bello A, Congy N, Sallusto F, et al. Anti-human leukocyte antigen immunization after early allograft nephrectomy. *Transplantation*. 2012;93(9). doi:10.1097/TP.0b013e31824b3720

4. Lucisano G, Brookes P, Santos-Nunez E, et al. Allosensitization after transplant failure: the role of graft nephrectomy and immunosuppression – a retrospective study. *Transplant International*. 2019;32(9):949-959. doi:10.1111/tri.13442

5. Goral S, Brukamp K, Ticehurst EH, et al. Transplant nephrectomy: histologic findings—a single center study. *Am J Nephrol*. 2014;40(5):491-498. doi:10.1159/000369865

6. Khakhar AK, Shahinian VB, House AA, et al. The impact of allograft nephrectomy on percent panel reactive antibody and clinical outcome. *Transplant Proc*. 2003;35(2):862-863. doi:10.1016/S0041-1345(02)04031-9

7. Augustine JJ, Woodside KJ, Padiyar A, Sanchez EQ, Hricik DE, Schulak JA. Independent of nephrectomy, weaning immunosuppression leads to late sensitization after kidney transplant failure. *Transplantation*. 2012;94(7):738-743. doi:10.1097/TP.0B013E3182612921

8. García Montemayor V, Agüera ML, Rabasco C, Navarro MD, Rodríguez Benot A, Aljama P. Development of Donor-Specific Anti-HLA Antibodies after Restarting Hemodialysis Due to Graft Failure. *Transplant Proc*. 2015;47(9):2626-2630. doi:10.1016/J.TRANSPROCEED.2015.10.018

9. Kosmoliaptsis V, Gjorgjimajkoska O, Sharples LD, et al. Impact of donor mismatches at individual HLA-A, -B, -C, -DR, and -DQ loci on the development of HLA-specific antibodies in patients listed for repeat renal transplantation. *Kidney Int*. 2014;86(5):1039-1048. doi:10.1038/ki.2014.106

10. Woodside KJ, Schirm ZW, Noon KA, et al. Fever, infection, and rejection after kidney transplant failure. *Transplantation*. 2014;97(6):648-653. doi:10.1097/01.TP.0000437558.75574.9C

11. Piatosa B, Rubik J, Grenda R. Is positive flow cytometric cross-match a risk factor for early cadaveric kidney graft dysfunction? *Transplant Proc*. 2006;38(1):53-55. doi:10.1016/j.transproceed.2005.12.081

12. Nimmo AMSA, McIntyre S, Turner DM, Henderson LK, Battle RK. The impact of withdrawal of maintenance immunosuppression and graft nephrectomy on HLA sensitization and calculated chance of future transplant. *Transplant Direct*. 2018;4(12). doi:10.1097/TXD.0000000000000848

13. Freist M, Bertrand D, Bailly E, et al. Management of Immunosuppression After Kidney Transplant Failure: Effect on Patient Sensitization. *Transplant Proc*. 2021;53(3):962-969. doi:10.1016/J.TRANSPROCEED.2020.10.009

14. Matignon M, Leibler C, Moranne O, et al. Anti-HLA sensitization after kidney allograft nephrectomy: changes one year post-surgery and beneficial effect of intravenous immunoglobulin. *Clin Transplant*. 2016;30(6):731-740. doi:10.1111/ctr.12743

15. Martin K, Cantwell L, Barraclough KA, et al. Prolonged immunosuppression does not improve risk of sensitization or likelihood of retransplantation after kidney transplant graft failure. *Transplant International*. 2021;34(11):2353-2362. doi:10.1111/TRI.13998

16. Schachtner T, Otto NM, Stein M, Reinke P. Transplantectomy is associated with presensitization with donor-reactive T cells and graft failure after kidney retransplantation: a cohort study. *Nephrol Dial Transplant*. 2018;33(5):889-896. doi:10.1093/ndt/gfy002

17. Lachmann N, Schönemann C, El-Awar N, et al. Dynamics and epitope specificity of anti-human leukocyte antibodies following renal allograft nephrectomy. *Nephrol Dial Transplant*. 2016;31(8):1351-1359. doi:10.1093/ndt/gfw041

18. Knight MG, Tiong HY, Li J, Pidwell D, Goldfarb D. Transplant nephrectomy after allograft failure is associated with allosensitization. *Urology*. 2011;78(2):314-318. doi:10.1016/j.urology.2011.02.068

19. Naini AE, Harandi AA, Daemi P, Kosari R, Gharavi M. Outcome of patients without any immunosuppressive therapy after renal allograft failure. *Saudi J Kidney Dis Transpl*. 2008;19(1):59-61. Accessed March 2, 2026. https://pubmed.ncbi.nlm.nih.gov/18087124/

20. Lenaers J, Christiaans M, Van Heurn E, Van Hooff H, Van Den Berg-Loonen E. Frequent but late donor-directed antibody formation after kidney transplantectomy within one month after grafting. *Transplantation*. 2006;81(4):614-619. doi:10.1097/01.tp.0000196726.91957.97

21. Billen EVA, Christiaans MHL, Lee J, Van Den Berg-Loonen EM. Donor-directed HLA antibodies before and after transplantectomy detected by the luminex single antigen assay. *Transplantation*. 2009;87(4):563-569. doi:10.1097/TP.0B013E3181949E37

22. Sener A, Khakhar AK, Nguan CY, House AA, Jevnikar AM, Luke PP. Early but not late allograft nephrectomy reduces allosensitization after transplant failure. *Can Urol Assoc J*. 2011;5(6). doi:10.5489/cuaj.10032

23. Adeyi OA, Girnita AL, Howe J, et al. Serum analysis after transplant nephrectomy reveals restricted antibody specificity patterns against structurally defined HLA class I mismatches. *Transpl Immunol*. 2005;14(1):53-62. doi:10.1016/j.trim.2005.01.001

24. Martínez Díaz M, Torío Ruíz A, Lorenzo González I, et al. Anti-HLA Antibodies After Precocious Transplantectomy by Vascular Thrombosis. *Transplant Proc*. 2018;50(2):546-549. doi:10.1016/j.transproceed.2018.01.002

25. Schrezenmeier E, Lehner LJ, Merkel M, et al. What happens after graft loss? A large, long-term, single-center observation. *Transplant International*. 2021;34(4):732-742. doi:10.1111/TRI.13834

26. Achinger SG, Ayus JC, Kumar A, Tsalatsanis A. Repeat Transplantation Rate of Patients Receiving Dialysis After Allograft Nephrectomy. *Kidney Med*. 2026;8(1). doi:10.1016/j.xkme.2025.101157

27. Leal R, Fragoso P, Venda J, et al. Prolonging calcineurin inhibitor therapy post kidney allograft failure: a prospective study. *Ren Fail*. 2025;47(1):2483386. doi:10.1080/0886022X.2025.2483386

1. [↑](#footnote-ref-1)
